# The chloroplast genome sequence and characteristic analysis of *Vitex negundo* var. *heterophylla* (Franch.) Rehder

**DOI:** 10.1080/23802359.2021.2005479

**Published:** 2021-11-29

**Authors:** Guangshun Zheng, Jianbo Li, Shu Diao

**Affiliations:** a National Permanent Scientific Research Base for Warm Temperate Zone Forestry of Jiulong Mountain in Beijing Experimental Center of Forestry in North China, Chinese Academy of Forestry, Beijing, China; b Research Institute of Subtropical Forestry, Chinese Academy of Forestry, Hangzhou, China

**Keywords:** Verbenaceae, *Vitex negundo*, chloroplast genome, phylogenetic analysis

## Abstract

*Vitex negundo* var. *heterophylla* (Franch.) Rehder is a common small shrub in northern China. In order to study the fine nectar characteristics and water and soil conservation characteristics of *V. negundo*, the analysis of chloroplast genome would provide theoretical basis for economic development and germplasm utilization of *V. negundo*. The chloroplast genome sequence (accession number MW366787) of *V. negundo* was accepted by high-throughput sequencing technology using a plant from Jiulongshan, Mentougou District, Beijing, China. The total length of the chloroplast genome is 154,438 bp, and the A, T, C and G content of the whole genome is 30.48, 31.26, 19.42, and 18.84%, respectively. The phylogenetic analysis of 16 Verbenaceae plants (including *V. negundo*) with *Arabidopsis thaliana* as the outgroup was carried out by the maximum likelihood method; and the result shows that *V. negundo* is relatively closed to *Vitex rotundifolia*.

*Vitex negundo* var. *heterophylla* (Franch.) Rehder is widely distributed in northern China and has been known as an excellent nectar plant. *Vitex negundo* var. *heterophylla* is a variant of *Vitex negundo*. The whole plant of *V. negundo* could be used medicinally to treat diseases such as asthma, stomach pain and tinea capitis (Meena et al. 2011). *V. negundo* is also widely accepted as the resource of health food due to the presence of biologically active ingredients, which could improve physical conditions in China. The seed of *V. negundo* contained a variety of phenolic compounds, which were evaluated the activities of antioxidant and anti-inflammatory. Most of the compounds showed strong 2,2′-Azinobis-(3-ethylbenzthiazoline-6-sulphonate) (ABTS) free radical scavenging activity. It was supported that *V. negundo* was a new potential medicinal plant for antioxidant and α-glucosidase inhibitor (Hu et al. 2015, 2016, 2017; Niu et al. 2020). The extract from *V. negundo* leaves has potential effects on immune hepatitis, and its mechanism may be related to free radical scavenging activity and inhibit release of NO (Huang et al. 2019; Zhou et al. 2011). In the extracts, the terpenoids and flavonoids could be used as potential anti-inflammatory candidates for the development of drugs or functional foods (Xu et al. 2019). At the same time, *V. negundo* has the ability to remove heavy metal pollution. When the initial cadmium concentration in the soil was 20 mg/kg (Tian et al. 2012). In addition, *V. negundo* is drought-tolerant and seen as a typical pioneer plant, widely distributed in various habitats, including hillsides, canyons, and rock crevices (Li et al. 2008). To exploit the resource of *V. negundo*, we sequenced its chloroplast genome for facilitating the process.

The fresh leaves of *V. negundo* were collected from Mentougou District, Beijing, China, and the samples were stored in Experimental Center of Forestry in North China (39.970°E, 116.096°N), Chinese Academy of Forestry. A specimen was deposited at Experimental Center of Forestry in North China (https://www.ncbi.nlm.nih.gov/nuccore/MW386998, contact person is Guangshun Zheng and email is guangshunzheng@163.com) under the voucher number jingtiao-001. After the DNA was extracted, a TruSeq DNA Sample Preparation Kit (Illumina, USA) and a Template Prep Kit (Pacific Biosciences, USA) were used to generate sequencing libraries. Genome sequencing was then performed by using the Illumina Novaseq platforms. A total of 21,497,706 reads (including 20,301,556 high-quality reads) were obtained. The chloroplast genome splicing results were obtained by using the software Mummer v3.1 (Kurtz et al. 2016) without the reference genome. The chloroplast splicing results were obtained by the above software. Mummer v3.1 was performed to determine the position relationship between contig and fill the gap between contigs. The results were corrected by using software Pilon v1.18 (Walker et al. 2014) to obtain the final chloroplast sequence. The online program Geseq web server (https://chlorobox.mpimp-golm.mpg.de/geseq.html) was performed to annotate the complete chloroplast genome sequence.

The assembled chloroplast genome of *V. negundo* has been submitted to the NCBI database (accession number MW366787). The *V. negundo* chloroplast genome is 154,438 bp in length, of which the GC content is 38.26%; the A, T, C and G content of the whole genome is 30.48, 31.26, 19.42 and 18.84%, respectively. The chloroplast genome distributed in a typical four-segment structure. It is found that the LSC region (large single copy region) is 85,146 bp, and the IRB region (inverted repeat B) and the other IR region IRA (inverted repeat A) is 25,686 bp, the SSC area (small single copy region) is 17,920 bp. And it is found the overall AT content is 61.7%, showing obvious AT preference. A total of 132 genes are encoded in *V. negundo* chloroplast genome, including 87 protein-coding, 37 tRNAs, and 8 rRNAs genes. The LSC region contains 62 protein-coding and 22 tRNAs genes, and the SSC region contains 14 protein-coding and 1 tRNA genes. The rRNA genes are only in the two IR regions with 4 types, respectively. Six protein-coding genes and seven tRNAs genes are only included in IR regions. Eighteen genes (including 6 tRNA genes) in the *V. negundo* chloroplast genome contains one or two introns, of which *ycf*3, *clp*P1 and *rps*12 contain two introns. Therefore, the position of *rps*12 is quite special. Its 5′-end is located in the LSC region, and the two 3′-ends are located in the IRA and IRB regions. The length of introns in the *V. negundo* chloroplast genome is different: *trnK-*UUU is with the longest intron of 2,507 bp, and *trnL*-UAA is with the shortest intron of 485 bp.

In order to confirm the phylogenetic position of *V. negundo*, another 15 complete chloroplast genome sequences from Verbenaceae plants and *Arabidopsis thaliana* (the outgroup) were downloaded from NCBI. The Maximum Likelihood method under MEGA7 (Kumar et al. 2016) in FastTree software was used to perform the phylogenetic tree. After the tree construction was completed, the reliability of the branch of the phylogenetic tree was verified (Bootstrap, 1000 replications). The result showed that *V. negundo* and *V. rotundifolia* in the genus *Vitex* were clustered together, indicating that their evolutionary relationship is closer than that of other Verbenaceae plants (Figure 1). The complete chloroplast genome of *V. negundo* is an important resource, which would help further research the population genetics, systematic geography, evolution, and conservation biology of Verbenaceae.

**Figure 1.**
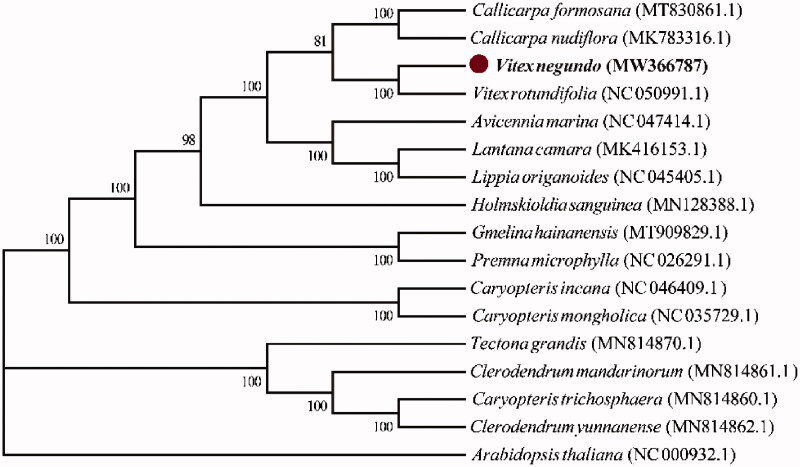
Evolutionary analysis of Verbenaceae species and *Arabidopsis thaliana* based on the whole chloroplast genome. The sequences were downloaded from NCBI, and the accession numbers are shown in the parentheses.

## Data Availability

The genome sequence data that support the findings of this study are openly available in GenBank of NCBI at [https://www.ncbi.nlm.nih.gov] (https://www.ncbi.nlm.nih.gov/) under the accession no. MW366787. The associated BioProject, SRA, and Bio-Sample numbers are PRJNA701363, SRR13684571, and SAMN17864120, respectively.
